# Risk Factors for Delirium Are Different in the Very Old: A Comparative One-Year Prospective Cohort Study of 5,831 Patients

**DOI:** 10.3389/fpsyt.2021.655087

**Published:** 2021-05-11

**Authors:** Justus Marquetand, Leonie Bode, Simon Fuchs, Florian Hildenbrand, Jutta Ernst, Roland von Känel, Soenke Boettger

**Affiliations:** ^1^Department of Consultation-Liaison Psychiatry and Psychosomatic Medicine, University Hospital Zürich, University of Zurich, Zurich, Switzerland; ^2^Department of Epileptology, Hertie-Institute for Clinical Brain Research, University of Tubingen, Tubingen, Germany; ^3^Department of Gastroenterology University Hospital Zürich, University of Zurich, Zurich, Switzerland; ^4^Institute of Nursing Science, University Hospital Zurich, University of Zurich, Zurich, Switzerland; ^5^University Hospital Zurich, University Zurich, Zurich, Switzerland

**Keywords:** delirium, very old, risk factors, comparison, prospective

## Abstract

**Background:** In an ever-aging society, health care systems will be confronted with an increasing number of patients over 80 years (“the very old”). Currently, knowledge about and recommendations for delirium management are often based on studies in patients aged 60 to 65 years. It is not clear whether these findings apply to patients ≥80 years.

**Aim:** Comparison of younger and older patients with delirium, especially regarding risk factors.

**Methods:** In this prospective cohort study, within 1-year, 5,831 patients (18–80 years: *n* = 4,730; ≥80: *n* = 1,101) with delirium were enrolled. The diagnosis of delirium was based on the Delirium Observation screening scale (DOS), Intensive Care Delirium Screening Checklist (ICDSC) and a DSM (Diagnostic and Statistical Manual)-5 construct of nursing instrument. Sociodemographic trajectories, as well as the relevant predisposing and precipitating factors for delirium, were assessed via a multiple regression analysis.

**Results:** The very old were more commonly admitted as emergencies (OR 1.42), had a greater mortality risk (OR 1.56) and displayed fewer precipitating risk factors for the development of a delirium, although the number of diagnoses were not different (*p* = 0.325). Predisposing factors were sufficient almost alone for the development of delirium in patients ≥ 80 years of age; in 18–80 years of age, additional precipitating factors had to occur to make a delirium possible.

**Conclusion:** When relevant predisposing factors for delirium are apparent, patients over 80 years of age require comparatively few or no precipitating factors to develop delirium. This finding should be taken into account at hospitalization and may allow better treatment of delirium in the future.

## Introduction

Delirium is the most common, acute neuropsychiatric disorder manifesting in abrupt and fluctuating disorders of consciousness, attention or cognition (e.g., concentration and memory) (1). The causes and risk factors for delirium are complex; indisputably, age is one of the major risk factors for the development of a delirium (2, 3).

Despite the rise of life expectancy in industrialized countries, and as a consequence increase in health care demands of aging patients, there is a lack of evidence of the characteristics of delirium in very old patients (≥80 years, also referred to as the “very old” or “very elderly”). It is unclear to what extent the delirium of this increasing number of patients differs from that in “younger” patients (18–80 years). The short and long-term socioeconomic and medical consequences of delirium are vast (4): Delirium is associated with higher health care costs, increased complications, mortality and loss of independence. Since developing delirium is associated with higher age and society in itself is getting older, there is a risk that the health care costs of delirium could exhaust the resources of future health care systems.

In addition to age, further risk factors for developing delirium can be divided into predisposing and precipitating factors (5–8). Predisposing factors exist prior to the development of delirium, e.g., dementia, substance addiction or gender. Precipitating factors, on the other hand, acutely cause delirium, e.g., infections, fever or surgeries. In general, the more predisposing factors exist in a patient, the fewer precipitating factors are necessary for the development of a delirium (9). Since aging in itself is associated with diseases and comorbidities or, predisposing factors, it is plausible that older patients have a higher risk of developing delirium than younger patients. As far as we know, the extent and differences vs. the younger patients have not been investigated. In general, previous studies commonly compared delirious vs. non-delirious patients, but the scope omits characteristics of delirious patients between age groups.

Therefore, in this study we compared very old delirious patients, ≥80 years, with younger delirious patients between 18 and 80 years. The cut-off-value ≥80 years was chosen due to several aspects: Since in the literature a distinction is made between old and very old patients and this cut-off value is repeatedly given at 80 years of age, it seemed reasonable to follow this cut-off value. Furthermore, the rate of multimorbidity (10), frailty (11, 12) and neurocognitive disorders (13) increases significantly from the age of 80 years, making a comparison with younger patients conclusive. The aim was to explore the distinction between these groups and to investigate the contribution of potential factors to inform future management studies or advanced care planning.

## Methods

### Study Design, Patients and Procedures

**Figure 1:**
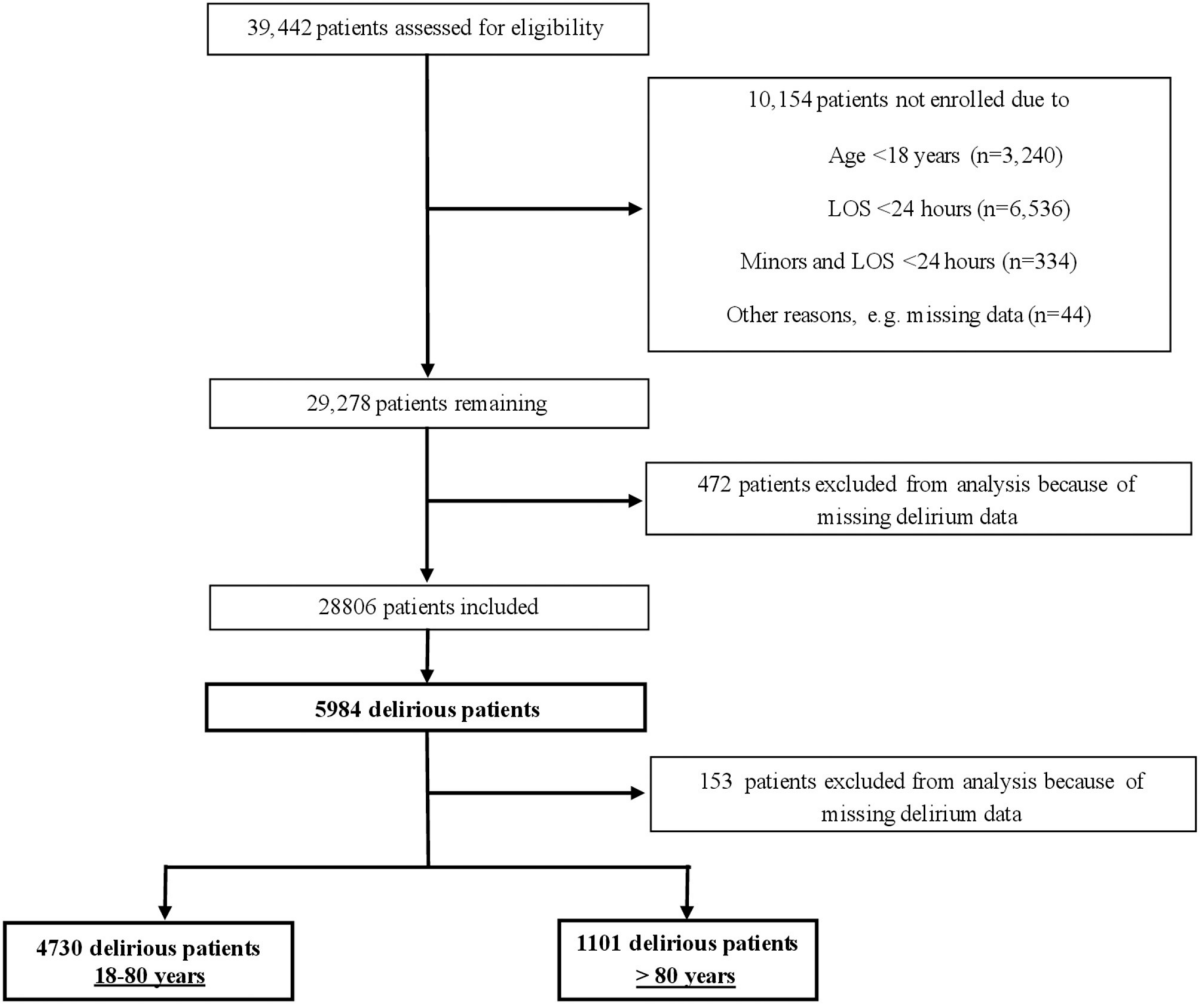
Screening-algorithm of the *Delir-Path*.

Between January 1st and December 31st 2014, a delirium detection initiative (DelirPath, **D**etect **E**valuate Contro**l I**npatient **R**isk factors, **P**revent **A**nd **T**reat **H**ospital Acquired Deliriums, Figure 1) at the University Hospital Zurich, a tertiary care center, prospectively assessed 39,442 patients for delirium. Patients were excluded if age was below 18 years, the length of stay (LOS) was below 1 day and missing data, including the electronic patient's assessment, leaving 28,806 eligible patients. Of these eligible patients, 5,984 (20.8%) had delirium. An additional 153 patients were excluded from this analysis due to partial incompleteness of available data. Of the remaining 5,831 patients, 1,101 (18.8%) were > 80 years and 4,730 (81.2%) were between 18 and 80 years old.

### Characterization of the Predisposing and Precipitating Factors for Delirium

Previously, several predisposing and precipitating factors for the development of delirium have been described. For the purpose of this study, predisposing and precipitating factors for the development of delirium were based on diagnostic clusters, according to the 10th revision of the International Statistical Classification of Diseases (ICD-10) (14) (Table 1), which is used in Switzerland. Furthermore, the ICD 10 provides uniform criteria and since the corresponding diagnoses are made by the respective specialists (e.g., the cardiologist diagnoses the heart disease), a high validity can be assumed.

**Table 1:** HDiagnostic clusters with their respective included diagnoses according to the International Statistical Classifications of Diseases and Related Health Problems 10th Revision (1CD-10).

	**ICD-10-Chapter**
Dementias/degenerative cerebral disorders	F00 Alzheimer's disease F01 Vascular dementias F02 Dementia due to elsewhere defined disorders F03 Neurodegenerative disorder G30 Alzheimer's disease G31-.0 Localized atrophies (frontal temporal dementia) G31-.1–2 Senile and alcohol-induced degenerations G31.8–9 Degenerations ned G32 Degenerations due to elsewhere defined disorders
Epilepsies	G40
Intracerebral hemorrhage	I61–62
Sepsis-related disorders	A40–41 Other sepsis, streptococcal B00.7 Herpetic sepsis R65 Systemic inflammatory response syndrome
Diseases of the genitourinary system	N18 Chronic renal failure
Diseases of the digestive system	K56 Paralytic ileus K65 Peritonitis K74 Liver cirrhosis K72 Liver failure including acute hepatitis
Neoplasm	C00-C69, C73-C97 C70-C72 neoplasm of the brain
Substance-induced	F10-F14
Hydrocephalus	G91
Brain edema	G93.6
Diseseas of the cardiovascular system	I10-I15 Arterial Hypertension I34-I37 Valvular heart disesase I42 Cardiomyopathy I70 Atherosclerosis I95 Arterial Hypotension
Diseases of the pulmonary system	J93 Pneumothorax
Syncope	R55
Malnutrition	E44

### Measurements and Diagnosis of Delirium

Since different delirium scales were used in normal wards and intensive care units, we used a set of three scales in total to measure delirium:

1) The Delirium Observation Screening Scale (DOS, cut-off ≥3) (15),
2) the Intensive Care Delirium Screening Checklist (ICDSC, cut-off ≥4) (16), and
3) a nursing instrument most recently validated by our group for the diagnosis of delirium, the Ergebnisorientiertes PflegeAssessment Acute-Care (ePA-AC) (17, 18) DSM-5-criteria (1), see also below.
  Given the circumstance that three different scales were used and the ePA-AC was not evaluated for delirium severity or subtype (hypoactive vs. hyperactive), we reduced the scales to whether delirium was present or not.

The DOS is a 13-item scale validated to indicate delirium according to DSM-IV criteria. Items include disturbances of consciousness (1), attention (2–4), thought processes (5 and 6), orientation (7 and 8), memory (9), psychomotor behavior (10, 11, and 13), and affect (12). Symptoms are rated on a scale (0–1) as not existent (0), sometimes to always existent (1), and unable to assess (-). The cut-off score for delirium is ≥3 and values were aggregated throughout recordings. This approach proved to be valid and correctly identified 91% of delirium diagnoses as determined by the consultation-liaison psychiatry service.

The ICDSC is a screening instrument with eight items based on the DSM-IV criteria specifically designed for the intensive care setting with two points: Absent or present. This scale was designed for patients with limited communication abilities such as intubated patients. The items include the assessment of 1 - consciousness (comatose, soporose, awake, or hypervigilant), 2 - orientation, 3 - hallucinations or delusions, 4 - psychomotor activity, 5 - inappropriate speech or mood, 6 - attentiveness, 7 - sleep-wake cycle disturbances and 8 - fluctuation of symptomatology. The maximum score is eight; scores of more than three indicate the presence of delirium. Each item is rated on the patient's behavior over the previous eight (15, 18).

The ePA-AC is a nursing instrument administered daily assessing mobility, personal care and dressing, feeding, elimination, cognition and alertness, communication and interaction, sleeping, breathing, pain, pressure ulcers and wounds (17).

On regular floors, patients ≥ 80 years were screened daily with DOS and ePA-AC. On intensive care units (ICU), ICDSC was conducted three times per day. Patients below 80 years were not routinely screened for delirium at hospital admission, but the delirium scales were applied in cases of clinical suspicion and a consultation psychiatry service was usually involved. DOS, ICDSC and ePA-AC were conducted by nursing staff and continued until remission of delirium was apparent. Nursing staff had been trained in a 4-h course with tests of achievement; In addition, literature research and eLearning were conducted. Further, the training was completed via case reports, lessons on epidemiology and characteristics of delirium, including the diagnostic criteria and approaches.

The chosen approach implementing the DOS, ICDSC and DSM-construct based on the ePA-AC was validated in the following manner: delirium diagnoses as determined by the gold-standard, the assessment by the consultation-liaison psychiatry service, were detected in 91%. Further, this construct was tested against the DOS and ICDSC and achieved perfect agreement (Cohen's κ 0.83, *p* < 0.001).

DOS, ICDSC and ePA-AC values as well as medical data was obtained from the electronic medical chart (Klinikinformationssystem, KISIM, CisTec AG, Zurich). This study was approved by the ethics committee of the Canton of Zurich (KEK-ZH-Nr. 2012-0263). A waiver of informed consent was obtained from the committee. Our reporting is in line with the STROBE (strengthening the reporting of observational studies in epidemiology)-statement (19).

### Statistical Methods

Data analysis, viewed in a highly simplified manner, involved two steps: (1) a descriptive description of sociodemographics and (2) a logistic regression of risk factors for delirium between the groups delirium 18–80 years vs. delirium ≥80 years. The analyses were performed with the Statistical Package for the Social Sciences (SPSS) version 25 and R statistical software version 3.5.0 for Windows.

Descriptive characteristics were summarized based on parametric properties using means and standard deviations or medians and interquartile ranges for continuous variables, and percentages for categorical variables. The data were tested with Shapiro-Wilk's test for distribution of normality. Inter-group differences for continuous variables were computed based on their parametric properties using Student's *t*-test and Mann-Whitney *U*-test, and Pearson's-χ^2^ test for categorical variables.

Then, simple logistic regressions were performed in order to determine the sociodemographic and clinical characteristics of delirium, as well as for the inclusion of medical clusters in the multiple regression analysis, with their respective odds ratios (OR) and corresponding confidence intervals (CIs). The multiple regression model was optimized with Cox-Snell's and Nagelkerke's r^2^ by omitting non-contributory cluster.

For all inferential tests, two-tailed tests were chosen and the significance level alpha (α) was set at p<0.05. the delirium construct based on DSM-5 was tested on its agreement with the validated approach - with a DOS cut-off ≥3 or ICDSC≥4 - with Cohen's κ as measure of concordance. The agreement was defined as >0.80 as perfect (20).

## Results

### Comparison of Characteristics of Delirious Patients

**Figure 2:**
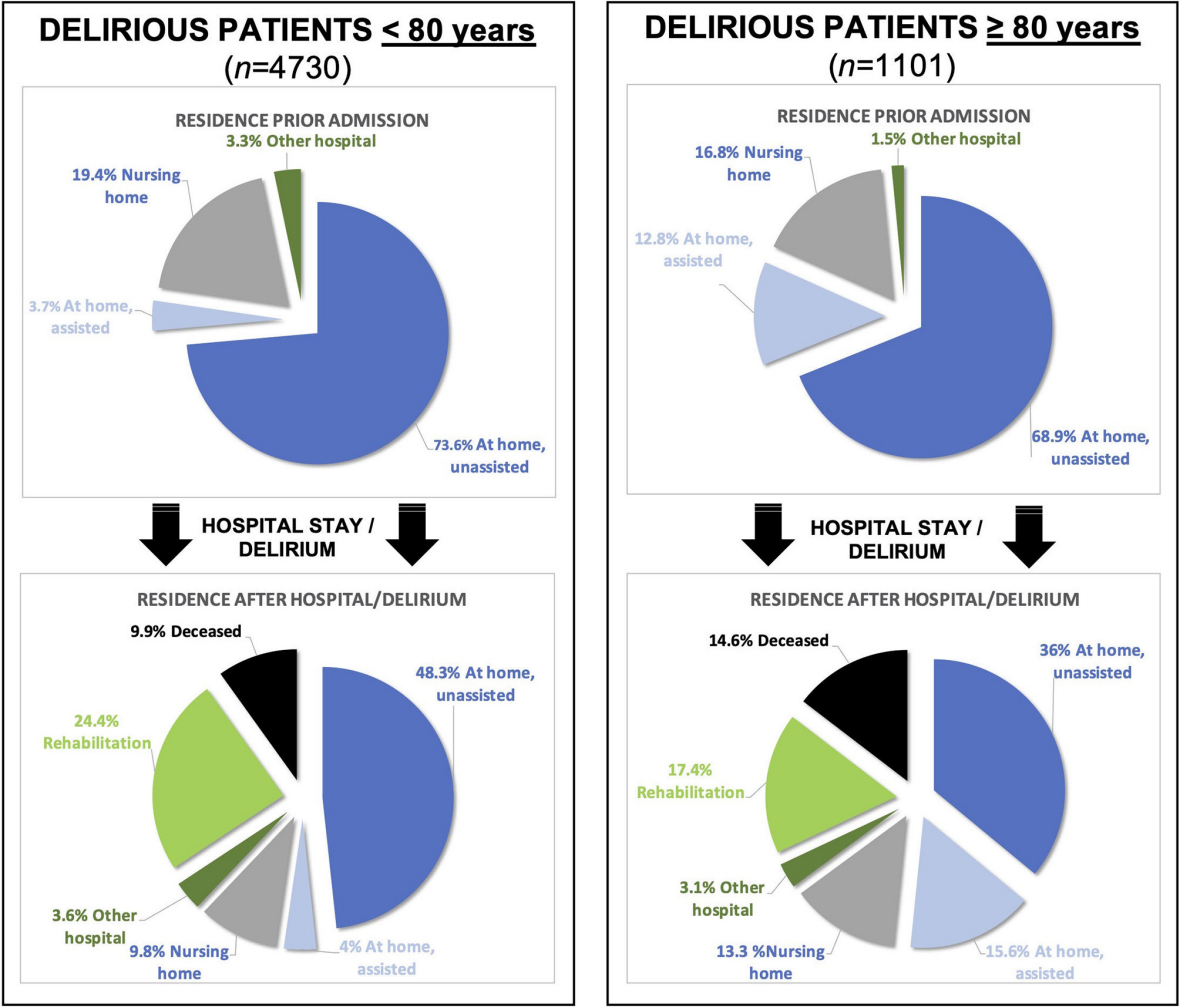
Graphical illustration (pie chart) of the residence before and after the hospital stay/delirium of the two groups. Note that delirious patients ≥ 80 years have a higher mortality and lose more often their independence.

The sociodemographic and medical characteristics of the delirious patients as well as the corresponding differences between age groups are displayed in Table 2 and Figure 2. There were group differences in terms of gender distribution, residence prior admission, admission mode, length of stay and residence after hospital. Within the very old (≥80), gender distribution was balanced, whereas in younger patients more men than women were delirious. Prior to admission, very old patients depended on assistance (OR 3.87) than living independently (OR 0.82), and were more likely to be admitted as emergencies (OR 1.42). Although emergent admissions might be due to greater comorbidity, neither the number of diagnoses nor involved organ systems were different between groups. The hospitalization of the very old was marginally shorter (10 vs. 11 days), however, they were more often transferred to a nursing home (OR 1.41), depended on assistance upon discharge at home (OR 4.4) or deceased (OR 1.56,). Conversely, transfers to rehabilitation were less common (OR 0.65).

Table 2Sociodemographic and medical characteristics of delirious patients.

**Table:** 

	**Patients** ≥***80 years*** (***n*** = 1,101)		**Patients** ***18–80 years*** (***n*** = 4,730)		
	**Mean (SD)**	**Median (IQR)**	**Mean (SD)**	**Median (IQR)**	***p***
Age in years	85 (4.2)	84 (6)	59 (15.2)	63 (21)	<0.001
Length of stay in days	12.4 (10.5)	10 (11)	16 (17.6)	11 (14)	<0.001
Number of Diagnoses	6.6 (5)	5 (6)	6.5(5.1)	5 (6)	0.325
Affected organ systems	3.9 (2.5)	3 (3)	3.8(2.6)	3 (3)	0.076
Operations	7 (10.4)	5 (7)	9.9 (10.4)	6 (10)	<0.001

**Table:** 

	**Percentage**	**Percentage**	***p***	**OR**	**CI**
**Gender**					
Male	50.2	62.8	<0.001	0.6	0.52–0.67
Female	49.8	37.2	<0.001	1.7	1.49–1.94
**Residence prior to admission**					
At home, unassisted	68.9	73.6	<0.001	0.82	0.71–0.95
At home, assisted	12.8	3.7	<0.001	3.87	3.08–4.86
Nursing home	16.8	19.4	0.072	0.9	0.72–1.01
Other hospital	1.5	3.3	0.001	0.44	0.26–0.72
**Admission**					
Emergency	66.5	58.7	<0.001	1.42	1.24–1.63
Elective	33.5	41.3	<0.001	0.76	0.66–0.87
**Residence after hospital/delirium**					
At home, unassisted	36	48.3	<0.001	0.6	0.53–0.69
At home, assisted	15.6	4	<0.001	4.4	3.55–5.47
Nursing home	13.3	9.8	0.001	1.41	1.16–1.71
Other hospital	3.1	3.6	0.44	0.87	0.59–1.25
Rehabilitation	17.4	24.4	<0.001	0.65	0.55–0.77
Deceased	14.6	9.9	<0.001	1.56	1.29–1.88

*SD, standard deviation; IQR, interquartile range; OR, odds ratio; CI, confidence interval*.

### Inter-group Differences of Predisposing Factors for Delirium

The differences between groups are listed in Table 3 and Figure 2. Corresponding regression coefficients [Exp (B)] at values >1 indicate higher risks (e.g., dementia) in the very old. Hence, dementia, arterial hypertension as well as hypotension, valvular heart disease, atherosclerosis and chronic renal failure predisposed the very old to develop a delirium. Conversely, in patients <80 years, these factors did less commonly lead to delirium. Predisposing factors such as liver cirrhosis, substance addiction or hydrocephalus increased the risk of delirium in patients <80 years, but comparatively less so in patients ≥80 years.

**Table 3:** Predisposing and precipitating risk factors.

***n=* 5,831**	**B (SE)**	**Exp (B)**	**CI**	**Sig**.
**Predisposing factors**				
Dementia	1.65 (0.13)	5.21	4.06–6.68	<0.001
Arterial hypertension	0.66 (0.07)	1.94	1.68–2.25	<0.001
Valvular heart disease	0.55 (0.1)	1.73	1.41–2.12	<0.001
Arterial hypotension	0.51 (0.25)	1.66	1.03–1.66	0.039
Chronic renal failure	0.61 (0.09)	1.56	1.56–2.19	<0.001
Atherosclerosis	0.4 (0.12)	1.5	1.18–1.89	0.001
Neoplastic disease (brain excluded)	–0.22 (0.09)	0.8	0.68–0.96	0.015
Malnutrition	−0.39 (0.16)	0.68	0.49–0.92	0.013
Cardiomyopathy	−0.52 (0.24)	0.6	0.38–0.94	0.028
Epilepsy	−0.61 (0.15)	0.54	0.4–0.73	<0.001
Neoplastic brain disease	−0.86 (0.44)	0.42	0.18–0.99	0.049
Hydrocephalus	−0.9 (0.29)	0.41	0.23–0.71	0.002
Substance-induced	−1.54 (0.2)	0.22	0.15–0.32	<0.001
Liver cirrhosis	−2.05 (0.72)	0.13	0.03–0.53	0.005
**Precipitating factors**				
Syncope	0.73 (0.35)	2.1	1.04–4.15	0.039
Intracranial hemorrhage	0.62 (0.18)	1.85	1.3–2.63	0.001
Sepsis/SIRS	−0.37 (0.13)	0.69	0.54–0.88	0.003
Liver failure	−0.71 (0.34)	0.49	0.25–0.96	0.037
Acute hepatitis	−1.05 (0.44)	0.35	0.15–0.82	0.016
Brain edema	−1.92 (0.73)	0.15	0.04–0.61	0.008
Constant	−1.84 (0.07)	0.16		

### Inter-group Differences of Precipitating Factors for Delirium

**Figure 3:**
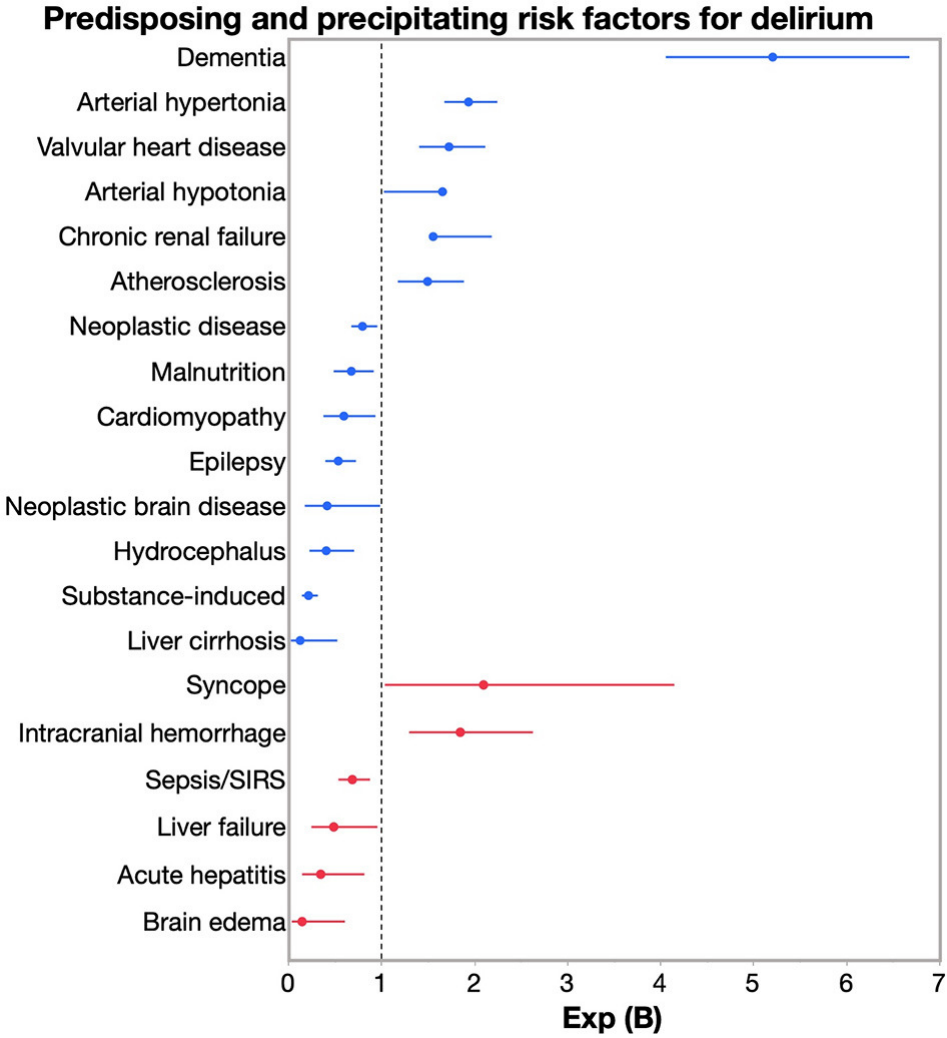
Risk for developing delirium: Exp (B), confidential intervals (CI) for well-known predisposing (blue) and precipitating (red) risk factors. Corresponding regression coefficients [Exp (B)] at values >1 indicate higher risks (e.g., dementia) in the very old.

The differences between groups are listed in Table 3 and Figures 2, 3. Syncope and intracranial hemorrhage were precipitating risk factors for developing delirium at ≥ 80 years, whereas in those <80 years, these factors were not as relevant. In patients <80 years, brain edema, acute hepatitis or liver failure were more common precipitating risk factors than in patients ≥ 80 years.

Only statistically significant results are reported. Factors frequently reported in the literature such as anemia, electrolyte disorder, or diabetes were analyzed, but did not reach the statistical significance level of *p* < 0.05.

## Discussion

### Summary of Main Findings

By comparing very old patients with younger ones between 18 and 80 years we found that delirium in patients ≥80 years occurs more commonly and is characterized by a complicated course and worse prognosis (e.g., mortality risk is 1.5 times higher). It is novel to compare delirious patients between age groups rather than non-delirious controls; hereby, the age factor can be better determined.

The prevalence of delirium in our sample was 20.8% (5,984 out of 28,806 patients), which concurs with numbers reported in the literature (21–23). Between groups, ≥ 80 vs. 18–80 years, the very old were more commonly admitted as emergencies, developed delirium more frequently and showed increased mortality. Comorbidities as measured with the number diagnoses were the same (*p* = 0.325), which might indicate a healthy survivor effect (24). Consequently, a delirium in patients ≥ 80 seems to have a stronger influence on the course during or after the hospital stay. It is consistent with the literature that delirium in the very old can be triggered by few or no precipitating factors, since apparently the presence of predisposing factors suffices (9, 21). These were dementias, cardiovascular diseases such as atherosclerosis, valvular heart disease or arterial hypertension. Further, the prognosis of delirium in the very old was dire, as they were more commonly transferred to a nursing home, were more commonly dependent on assistance at home, less commonly transferred to rehabilitation, or deceased. Interestingly, their hospitalization was marginally shorter, but this might also reflect earlier transfer and higher mortality rate.

### Comparison With the Existing Literature

To our knowledge, there are no studies to date that directly compare different age groups of delirium patients, especially not in patients ≥80 years.

However, there are few comparative studies that have examined how the type and severity of delirium differ between children (0–17 years), adults (18–65 years) or elderly patients (66–91 years) (25–27). Grover and colleagues (27) described that adults and elderly patients did not differ significantly in severity or type of delirium: 321 delirium patients, 245 adults (18–64 years), and 76 elderly patients (≥ 65 years) were compared regarding the prevalence of underlying etiologies: In the elderly group, decompensations of cardiovascular disease were more common; in contrast, substance abuse or intoxications were more common in adult delirium patients. Consistent with these findings, the logistic regression in our study shows that substance abuse in those <80 years of age and cardiovascular disease in those >80 years of age have a higher risk of developing delirium. In addition, there are studies examining the effect of age in alcohol delirium by forming subgroups in decades (e.g., 20–30 years, 30–40 years), but unfortunately no patient groups >80 years and only in alcohol withdrawal delirium. One meta-analysis compared symptoms in delirious pediatric and adult patients (28), but this study does not reflect the theme of our study in the very elderly.

Furthermore, there are studies comparing delirious patients of different specialties (29), but studies comparing age groups, especially regarding risk factors, do not exist.

### Implications

The causes, manifestations and outcomes of delirium vary with age; although this may seem trivial, in this study of the very old, ≥ 80 years of age, admission mode, predisposing and precipitating factors for delirium and outcome were very different and not advantageous. The results of this study can be used as possible starting points for future management studies, as well as advanced care planning and, once again, illustrate that delirium is a common and potentially life-threatening condition.

### Strengths and Limitations

Clear strengths of this study are the overall (1) large group sizes and (2) prospective data collection and (3) extensive description of sociodemographic, medical and clinical characteristics of delirious patients. A novelty in this study is the comparison between two age groups of delirious patients, which leads to a better determination of the age factor. The differences in group sizes (4,730 vs. 1,101 patients) can be considered problematic, potentially having led to disproportionate power in 18–80-year-olds; however, the different group sizes represent the natural demographic age distribution. In addition, the severity of diseases was not characterized, as this is difficult to statistically represent or operationalize. A major limitation of this study may represent the dichotomization of the variable age, which, while providing good contrast between groups, may also distort the results. Future studies on the variable age are necessary to further investigate this effect. The collected data is from 2014 and may not be fully generalizable due to improved delirium prevention in recent years. The administration of any medication (e.g., benzodiazepines, antipsychotics) was not recorded, as this was not methodologically possible. Our patient population was representative for a tertiary care center, generalizability to other health care facilities is limited. Since patients > 80 years of age were routinely screened for delirium, but patients <80 years of age were not, there is a possibility that delirium is underdiagnosed in this age group, leading to biased results. Future studies are required to confirm these findings.

## Conclusions

Delirium in very old patients, i.e., those ≥ 80 years, is different from delirium in the general hospital population. The very old are at high risk for developing delirium. When relevant predisposing factors for delirium become apparent, the very old require only few or no precipitating factors for the development of delirium. This should be accounted for on admission and may allow better screening for and management of delirium in this age group in the future.

## Location of Conduction

Between January 1st and December 31st 2014, a delirium detection initiative (DelirPath, **D**etect **E**valuate Contro**l I**npatient **R**isk factors, **P**revent **A**nd **T**reat **H**ospital Acquired Deliriums, Figure 1) at the University Hospital Zurich, a tertiary care center, prospectively assessed 39,442 patients for delirium.

## Data Availability Statement

The raw data supporting the conclusions of this article will be made available by the authors, without undue reservation.

## Ethics Statement

The studies involving human participants were reviewed and approved by ethics committee of the Canton of Zurich (KEK-ZH-Nr. 2012-0263). The ethics committee waived the requirement of written informed consent for participation.

## Author Contributions

JM analyzed clinical and diagnostic data and drafted and revised the manuscript. LB acquired clinical and diagnostic data and revised the manuscript. SF acquired clinical and diagnostic data and revised the manuscript. FH, JE, and RK analyzed clinical and diagnostic data and revised the manuscript. SB conceptualized the study, acquired and analyzed clinical and diagnostic data, and revised the manuscript. All authors contributed to the article and approved the submitted version.

## Conflict of Interest

The authors declare that the research was conducted in the absence of any commercial or financial relationships that could be construed as a potential conflict of interest.
