# Corrigendum: Risk Factors for Delirium Are Different in the Very Old: A Comparative One-Year Prospective Cohort Study of 5,831 Patients

**DOI:** 10.3389/fpsyt.2022.885734

**Published:** 2022-03-21

**Authors:** Justus Marquetand, Leonie Bode, Simon Fuchs, Florian Hildenbrand, Jutta Ernst, Roland von Känel, Soenke Boettger

**Affiliations:** ^1^Department of Consultation-Liaison Psychiatry and Psychosomatic Medicine, University Hospital Zürich, University of Zurich, Zurich, Switzerland; ^2^Department of Epileptology, Hertie-Institute for Clinical Brain Research, University of Tubingen, Tubingen, Germany; ^3^Department of Gastroenterology University Hospital Zürich, University of Zurich, Zurich, Switzerland; ^4^Institute of Nursing Science, University Hospital Zurich, University of Zurich, Zurich, Switzerland; ^5^University Hospital Zurich, University Zurich, Zurich, Switzerland

**Keywords:** delirium, very old, risk factors, comparison, prospective

An author name was incorrectly spelled as “Roland von Kaenel.” The correct spelling is “Roland von Känel.”

The authors apologize for this error and state that this does not change the scientific conclusions of the article in any way. The original article has been updated.

## Publisher's Note

All claims expressed in this article are solely those of the authors and do not necessarily represent those of their affiliated organizations, or those of the publisher, the editors and the reviewers. Any product that may be evaluated in this article, or claim that may be made by its manufacturer, is not guaranteed or endorsed by the publisher.

